# Supplementary material for: Computational Drug Repurposing Algorithm Targeting TRPA1 Calcium Channel as a Potential Therapeutic Solution for Multiple Sclerosis
Source: Pharmaceutics. 2019 Sep 2;11(9):446. doi: 10.3390/pharmaceutics11090446 (PMC6781306; doi:10.3390/pharmaceutics11090446)
Supplement: Supplementary file 1 [file pharmaceutics-11-00446-s001.pdf]

pharmaceutics

Supplementary Materials: Computational Drug Repurposing Algorithm Targeting TRPA1 Calcium Channel as a Potential Therapeutic Solution for Multiple Sclerosis

Dragos Paul Mihai, George Mihai Nitulescu *, George Nicolae Daniel Ion, Cosmin Ionut Ciotu, Cornel Chirita, and Simona Negres

Table S1. Descriptive statistics for pIC50 and druglikeness-related descriptors for the TRPA1 inhibitors set.

Descriptor	Range	Minimum	Maximum	Mean ± SD
pIC_{50} (M)	4.48	4.52	9.00	6.57 ± 1.01
ALogP	8.28	−0.71	7.57	4.02 ± 1.34
Molecular weight	482.03	175.10	657.13	389.70 ± 101.73
Polar surface area	193.59	17.82	211.41	82.80 ± 40.83
Rotatable bonds	12	1	13	5.01 ± 2.08
Hydrogen bonds acceptors	8	0	8	2.92 ± 1.46
Hydrogen bonds donors	3	0	3	1.06 ± 0.54

SD – standard deviation.

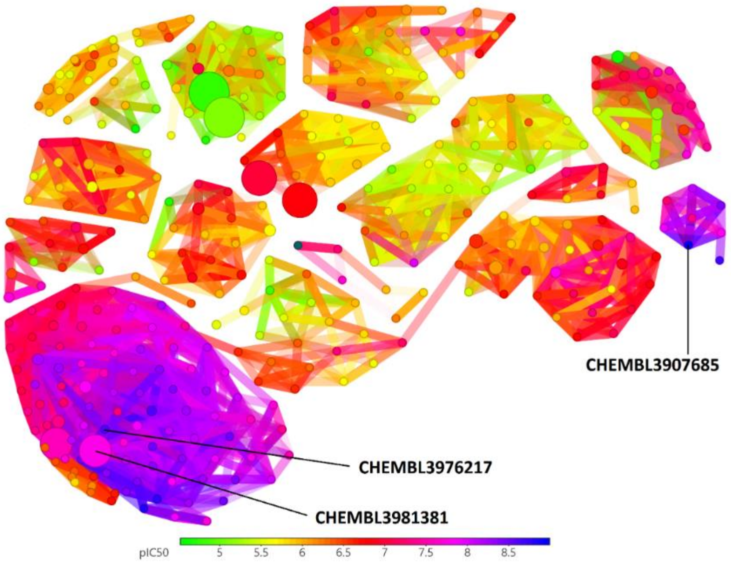

Figure S1. Diagram of similarity/activity cliffs based on flexophores with 80% similarity within TRPA1 inhibitors. Larger dots indicate the presence of an activity cliff.

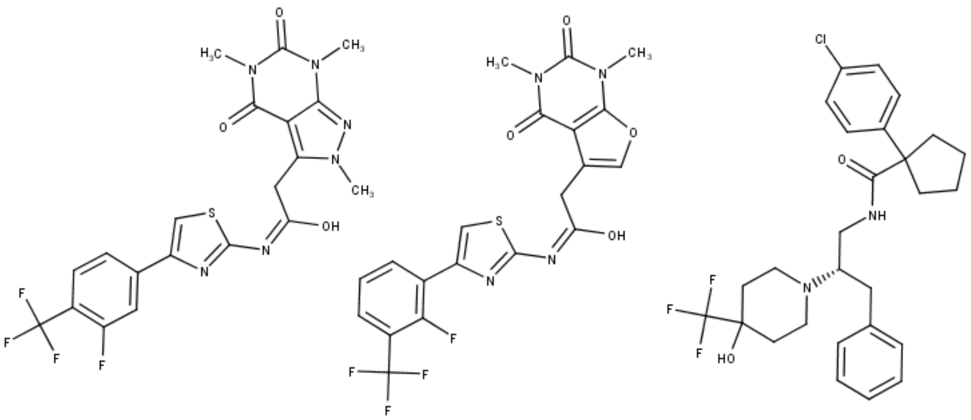

CHEMBL3981381
pIC50 = 7.79

CHEMBL3976217
pIC50 = 8.65

CHEMBL3907685
pIC50 = 8.99

Figure S2. Representative structures for similarity/activity cliffs analysis of TRPA1 inhibitors.

Table S2. Highest similarity pairs between TRPA1 inhibitors and screened drugs based on flexophore descriptors data mining procedure.

Entry	TRPA1 inhibitors (ChEMBL ID)	Repurposing dataset (DrugBank ID)	Similarity
1	CHEMBL3298238	DB08135	0.9832
2	CHEMBL3220230	DB08561	0.9696
3	CHEMBL3220228	DB08561	0.9614
4	CHEMBL593902	DB07311	0.9553
5	CHEMBL3297780	DB01065	0.9533
6	CHEMBL3220448	DB08561	0.9509

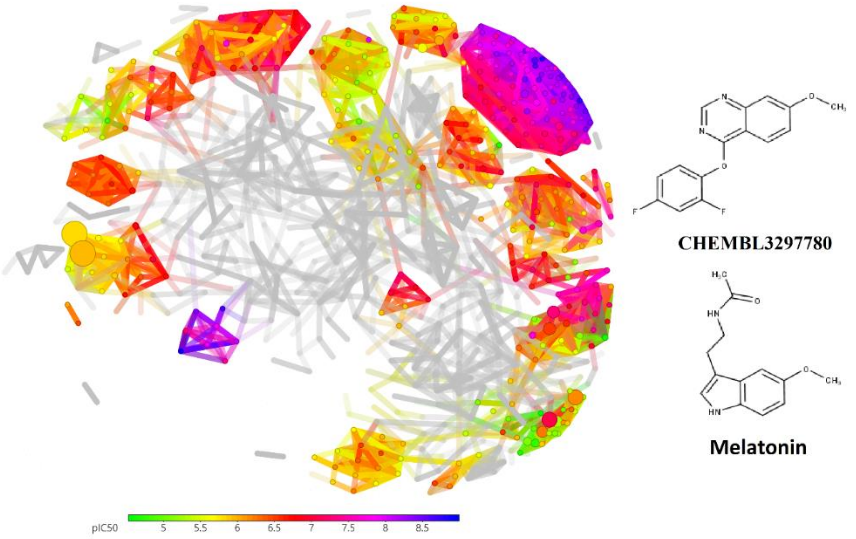

Figure S3. Diagram of similarity/activity cliffs based on flexophores with 80% similarity threshold for merged TRPA1 inhibitors dataset (colored dots) and similar DrugBank entries (grey dots).

Table S3. Binary classification model evaluation metrics.

Set	Sensitivity	Specificity	Accuracy	ROC AUC	F1 score
Calibration	0.840	0.875	0.857	0.891	0.859
Validation	0.794	0.826	0.813	0.874	0.783
Global	0.817	0.851	0.835	0.890	0.821

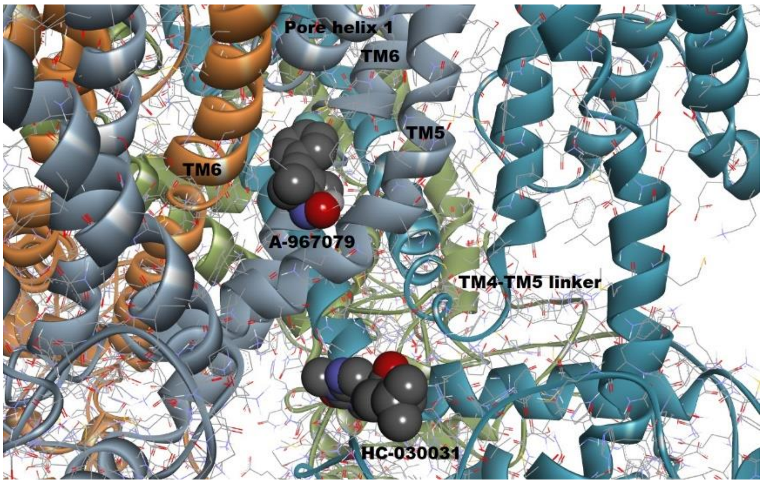

Figure S4. Conformations of A-967079 and HC-030031 generated by induced fit molecular docking simulations.

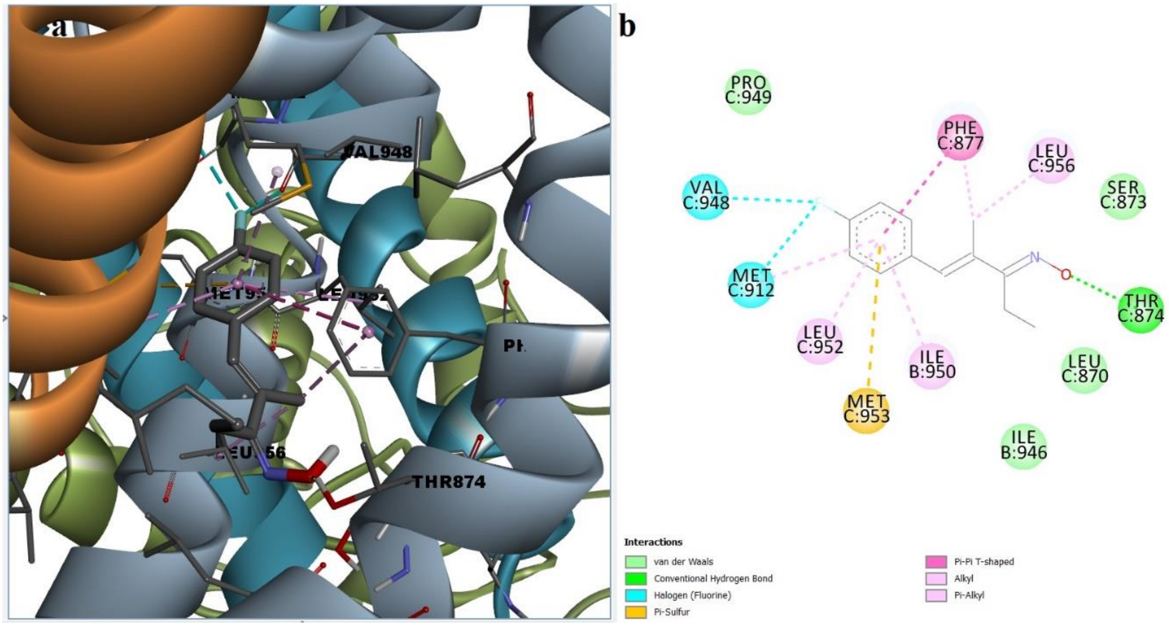

Figure S5. a – 3D binding conformation of A-967079 into the binding site; b – 2D diagram of protein-ligand interactions between TRPA1 and A-967079.

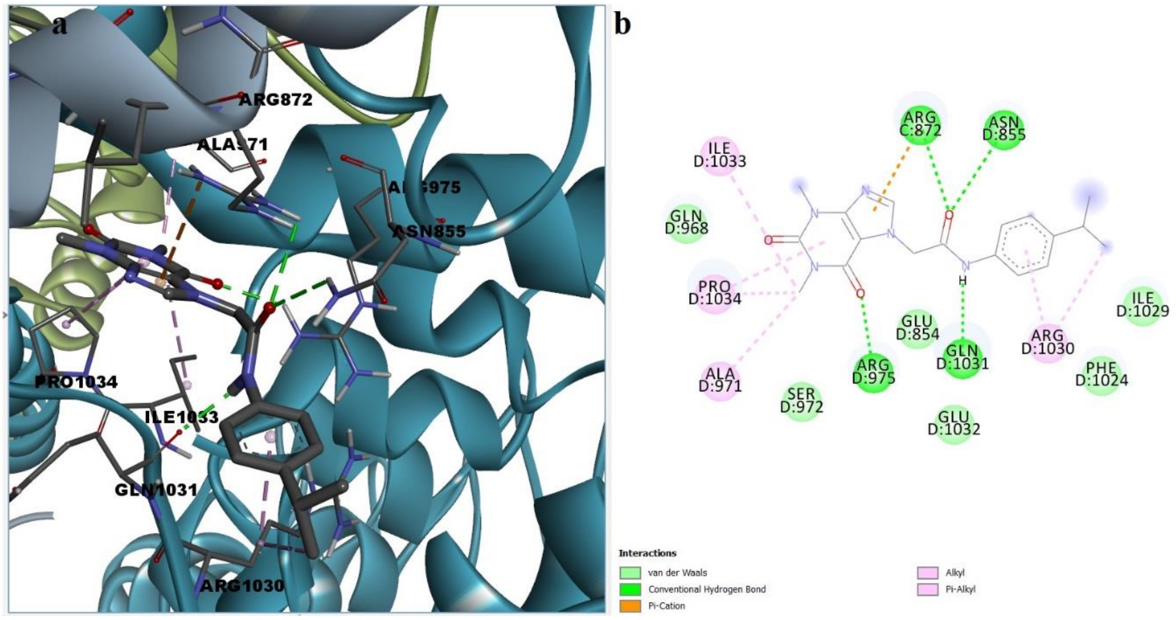

Figure S6. a – 3D binding conformation of HC-030031 into the binding site; b – 2D diagram of protein-ligand interactions between TRPA1 and HC-030031.

Table S4. Quality assessment parameters of proposed binary logistic regression model.

Subset	Sensitivity	Specificity	Accuracy	ROC AUC	F1 index
Calibration	0.905	0.934	0.920	0.975	0.916
Validation	0.932	0.953	0.943	0.983	0.943
Global	0.919	0.944	0.932	0.978	0.929
Decoy	-	-	0.921	-	-

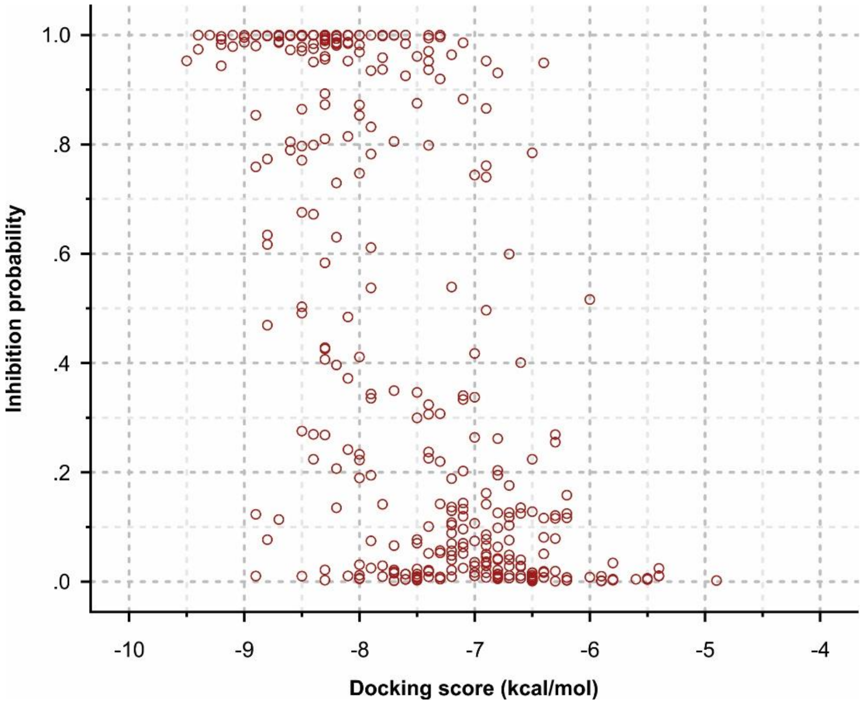

Figure S7. Scatter plot of TRPA1 inhibition probability against the predicted binding energy (ΔG) for all TRPA1 inhibitors (weak, moderate, and strong) estimated by the global prediction model.

Table S5. Top 10 primarily ranked potential TRPA1 inhibitors based on the binary logistic regression equation used as a global prediction model.

DrugBank ID	Generic name	Drug groups	Biological activity	Score	Activity class	pIC50pred (M)	ΔG (kcal/mol)	P
DB05137	lobeline	I	nicotinic agonist	3	1	10.89	−7.3	1.00000
DB11518	flunixin	V	NSAID	3	1	10.81	−7.0	1.00000
DB02331	none	E	genome polyprotein	4	1	10.60	−8.4	1.00000
DB01082	streptomycin	A, V	antibiotic	3	1	10.66	−7.5	1.00000
DB13374	vincamine	E	vasodilator	3	1	10.54	−7.1	1.00000
DB04204	none	E	PTPN1	3	1	9.93	−6.4	1.00000
DB13953	estradiol benzoate	A, I, V	sex steroid	3	1	9.36	−8.3	1.00000
DB11629	laropiprant	A, I, W	selective DP1 antagonist	3	1	9.38	−7.3	1.00000
DB03970	none	E	beta-lactamase inhibitor	4	1	9.13	−7.0	0.99999
DB11979	elagolix	A, I	GnRh antagonist	4	1	8.97	−7.9	0.99999

Score – data mining score; pIC_{50}*pred* – MLR predicted pIC_{50}; ΔG – predicted binding energy (kcal/mol); P – probability of TRPA1 inhibition; I – investigational; V – veterinary approved; E – experimental; A – approved; W- withdrawn.

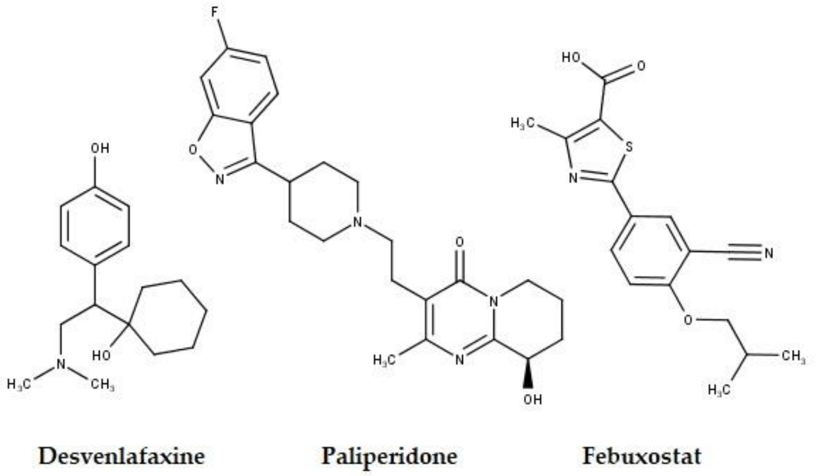

Figure S8. Chemical structures of three commercially available screening hits.

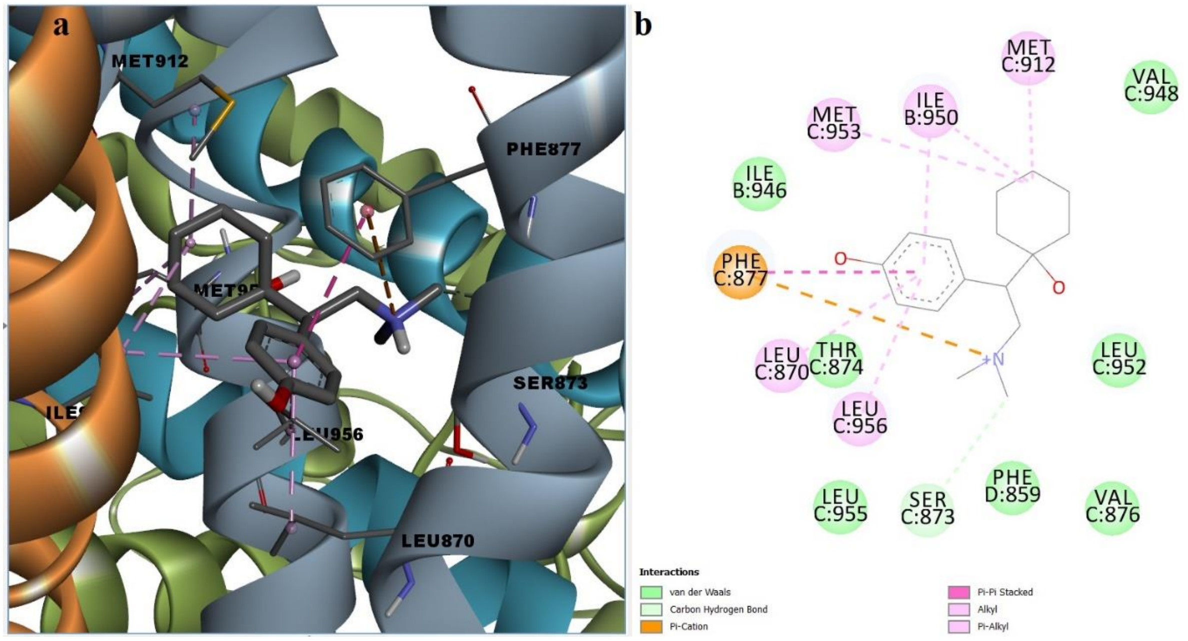

Figure S9. a – 3D binding conformation of desvenlafaxine into putative A-967079 binding site; b – 2D diagram of protein-ligand interactions between TRPA1 and desvenlafaxine.
